# Complete Genome Sequence of *Citrobacter freundii* Myophage Michonne

**DOI:** 10.1128/genomeA.01134-15

**Published:** 2015-10-01

**Authors:** Christopher L. Bernal, Victoria E. Berkowitz, Jesse L. Cahill, Eric S. Rasche, Gabriel F. Kuty Everett

**Affiliations:** Center for Phage Technology, Texas A&M University, College Station, Texas, USA

## Abstract

*Citrobacter freundii* is a Gram-negative opportunistic pathogen that causes dangerous infections such as neonatal meningitis. *C. freundii* also harbors antibiotic resistance, making phages infecting this host valuable tools. Here, we announce the complete genome of the *C. freundii* FelixO1-like myophage Michonne and describe its notable features.

## GENOME ANNOUNCEMENT

*Citrobacter freundii* is found in soil, water, sewage, and the intestinal tracts of animals and humans*.* It acts as an opportunistic pathogen, causing urinary tract and blood infections, often nosocomial in nature, and is a major cause of neonatal meningitis (1). *C. freundii* also exhibits high antimicrobial resistance to penicillins and third-generation cephalosporins (2), emphasizing the need for alternative treatments like bacteriophages. The novel FelixO1-like myophage Michonne, described here, may be useful in the biocontrol of *C. freundii*.

Bacteriophage Michonne was isolated from a sewage sample collected in College Station, TX. Phage DNA was sequenced in an Illumina MiSeq 250-bp paired-end run with a 550-bp insert library at the Genomic Sequencing and Analysis Facility at the University of Texas (Austin, TX, USA). Quality controlled, trimmed reads were assembled to a single contig of circular terminally redundant assembly at 56.3-fold coverage using SPAdes version 3.5.0 (3). The contig was confirmed to be complete by PCR using primers that faced the upstream and downstream ends of the contig. Products from the PCR amplification of the junctions of concatemeric molecules were sequenced by Sanger sequencing (Eton Bioscience, San Diego, CA). Genes were predicted using GeneMarkS (4) and corrected using software tools available on the Center for Phage Technology (CPT) Galaxy instance (https://cpt.tamu.edu/galaxy-public/). Morphology was determined using transmission electron microscopy performed at the Texas A&M University Microscopy and Imaging Center.

Michonne is a FelixO1-like myophage with a 90,000-bp genome, a coding density of 90%, and a G+C content of 38.9%. It encodes 143 coding sequences (CDSs), 36 of which were functionally annotated using BLASTp and InterPro Scan analysis (5, 6). Michonne shares 48.8% nucleotide sequence identity across the genome with FelixO1 (NC_005282), as determined by Emboss Stretcher (7). It contains 25 tRNAs compared to the 22 tRNA genes identified in FelixO1. CoreGenes analysis shows that 117/143 (81.8%) of the proteins encoded by Michonne are homologs of those encoded by FelixO1 (8). Michonne has a nearly identical G+C composition to FelixO1 (39.0%), a comparable coding density (90.6%), and appears to share its characteristic of possessing only a TerL terminase subunit (9). Michonne was found to contain only 3 homing endonucleases (compared to the 6 identified in FelixO1). For annotation purposes, it has been opened to the *rIIa* gene by precedent. Other related phages include *Enterobacteria* phage WV8 (NC_012749) (48.6%), *Erwinia* phage phiEa104 (NC_015292) (48.4%), and *Citrobacter* phage Moogle (KM236239) (83.6%) (10–12).

The genome of Michonne has FelixO1-like genes, including those whose products are involved in morphogenesis, biosynthesis, DNA replication, and lysis. The two phages differ mostly in hypothetical proteins of unknown function. The tape measure protein of Michonne is preceded by a tail assembly protein that uses a translational frameshift to the −1 reading frame to achieve a second product, as is seen in many *Caudovirales* (13). Lysis genes identified include a putative class-III holin, a soluble lysozyme, and inner and outer spanin genes (14, 15).

### Nucleotide sequence accession number.

The genome sequence of phage Michonne was contributed to GenBank with the accession number KT001916.
